# Personalized Medicine in Pancreatic Cancer: The Promise of Biomarkers and Molecular Targeting with Dr. Michael J. Pishvaian

**DOI:** 10.3390/cancers16132329

**Published:** 2024-06-26

**Authors:** Viviana Cortiana, Rabab Hunaid Abbas, Harshal Chorya, Jade Gambill, Diksha Mahendru, Chandler H. Park, Yan Leyfman

**Affiliations:** 1 Department of Medical and Surgical Sciences (DIMEC), University of Bologna, 40126 Bologna, Italy; 2 Tbilisi State Medical University, 0186 Tbilisi, Georgia; 3 Medical College Baroda, Vadodara 390018, India; 4 Parker University, Dallas, TX 75229, USA; 5 Global Remote Research Scholars Program, St. Paul, MN 55101, USA; 6 Norton Cancer Institute, Louisville, KY 40202, USA; chandler.park@louisville.edu; 7 Icahn School of Medicine at Mount Sinai South Nassau, Oceanside, NY 11572, USA; yan.leyfman@mssm.edu

**Keywords:** pancreatic cancer, biomarkers, early detection, personalized medicine, next-generation sequencing (NGS), genetic mutations, germline testing

## Abstract

**Simple Summary:**

Pancreatic cancer, with its increasing incidence, is predicted to become the second deadliest solid tumor by 2040, urging for improved diagnostic and treatment strategies. Despite advancements, the five-year survival rate is about 14%, decreasing further with metastasis. This review examines the potential of biomarkers for early detection, personalized treatment, and disease monitoring. Molecular classification based on genetic mutations and protein markers aids treatment decisions and improves outcomes. Ongoing clinical trials investigate targeted therapies like CLAUDIN 18.2 and Claudin 18.1 inhibitors. Next-generation sequencing (NGS) enables comprehensive genomic analysis, allowing tailored therapies. However, challenges include limited awareness and uptake of biomarker-guided therapies, necessitating continued research.

**Abstract:**

Pancreatic cancer, with its alarming rising incidence, is predicted to become the second deadliest type of solid tumor by 2040, highlighting the urgent need for improved diagnostic and treatment strategies. Despite medical advancements, the five-year survival rate for pancreatic cancer remains about 14%, dropping further when metastasized. This review explores the promise of biomarkers for early detection, personalized treatment, and disease monitoring. Molecular classification of pancreatic cancer into subtypes based on genetic mutations, gene expression, and protein markers guides treatment decisions, potentially improving outcomes. A plethora of clinical trials investigating different strategies are currently ongoing. Targeted therapies, among which those against CLAUDIN 18.2 and inhibitors of Claudin 18.1, have shown promise. Next-generation sequencing (NGS) has emerged as a powerful tool for the comprehensive genomic analysis of pancreatic tumors, revealing unique genetic alterations that drive cancer progression. This allows oncologists to tailor therapies to target specific molecular abnormalities. However, challenges remain, including limited awareness and uptake of biomarker-guided therapies. Continued research into the molecular mechanisms of pancreatic cancer is essential for developing more effective treatments and improving patient survival rates.

## 1. Introduction

Pancreatic cancer, a malignancy originating in the pancreas, is a critical health concern and a challenging disease due to its aggressive nature and often asymptomatic early stages [1]. The pancreas, located in the upper abdomen, plays a crucial role in digestion and hormone regulation. However, people often detect the disease only in advanced stages, partly due to its lack of early and specific symptoms [2]. There are two primary types of pancreatic cancer: exocrine and neuroendocrine. Exocrine pancreatic cancer, which accounts for over 90% of cases, stems from the exocrine cells that form the pancreas’s glands and ducts, with adenocarcinoma being the most prevalent subtype, though others like squamous cell and colloid carcinomas exist. On the other hand, neuroendocrine pancreatic cancer originates from the endocrine cells producing hormones like insulin and glucagon [3,4]. Neuroendocrine pancreatic cancer is a rarer subtype of pancreatic cancer and, unfortunately, it remains challenging for providers to treat both the tumor growth as well as correlated symptoms, with surgery still persisting as the main form of treatment for this subtype [5]. For the purpose of this review, however, we will be focusing on the exocrine subtype of pancreatic cancer. Symptoms of pancreatic cancer include severe abdominal pain, weight loss, jaundice, dark urine, clay stools, itching, appetite loss, blood clots, fatigue, and new-onset diabetes. Diagnosis typically involves imaging tests and a biopsy [6]. Despite medical advancements, pancreatic cancer’s survival rates remain low, necessitating treatment modalities like surgery, chemotherapy, radiation, or a combination, highlighting the ongoing challenge this disease poses to healthcare and the urgent need for further research in the field.

### 1.1. Pancreatic Cancer Epidemiology

Epidemiologically, pancreatic cancer incidence has steadily increased over the years. It ranks as the twelfth most prevalent cancer among males, the eleventh most prevalent cancer among females, and is currently the seventh most significant contributor to cancer-related mortality globally [7]. The American Cancer Society (ACS) predicts that 66,440 individuals in the US will receive a pancreatic cancer diagnosis in 2024. Of these, 34,530 will be men, and 31,910 will be women. Tragically, the disease will claim the lives of approximately 51,750 people, including 27,270 men and 24,480 women. Researchers estimated that the average lifetime risk of developing pancreatic cancer is one in 56 men and one in 60 women. The incidence and mortality rates are nearly identical, highlighting the aggressive nature of this disease and the urgent need for more effective therapeutic strategies [8]. By 2040, projections indicate that pancreatic cancer will become the second most common cause of cancer-related mortality in the United States [9]. This highlights the urgent need to enhance our understanding and treatment of this fatal disease. These data highlight the pressing necessity for more awareness, research, and innovative approaches to improve pancreatic cancer care.

### 1.2. Current Standards of Care

The current standards of care for pancreatic cancer include surgery, chemotherapy, and radiation therapy. However, these conventional treatments often offer limited benefits, particularly in the advanced stages of the disease. Among the established treatment regimens, FOLFIRINOX and gemcitabine plus nab-paclitaxel have emerged as cornerstones in the therapeutic armamentarium. FOLFIRINOX is a combination chemotherapy regimen that includes fluorouracil, leucovorin, irinotecan, and oxaliplatin. It has been shown to work in certain patient groups. Similarly, gemcitabine plus nab-paclitaxel has emerged as a viable treatment option for patients with advanced pancreatic cancer [10]. These regimens, while offering incremental improvements in patient outcomes, fail to achieve meaningful long-term survival benefits. Therefore, there is an urgent need for novel therapeutic approaches that address the underlying biology of the disease and offer improved outcomes for patients. In a pivotal phase 3 trial, NAPOLI 3 compared NALIRIFOX with nab-paclitaxel and gemcitabine as initial therapies for metastatic pancreatic ductal adenocarcinoma (mPDAC). The study, conducted globally across 187 sites, demonstrated a significant improvement in median overall survival with NALIRIFOX (11.1 months) compared to nab-paclitaxel-gemcitabine (9.2 months). Both regimens exhibited manageable safety profiles, with similar rates of grade 3 or higher adverse events and treatment-related deaths. These results position NALIRIFOX as a potential standard of care for first-line treatment of mPDAC [11].

**Figure 1:**
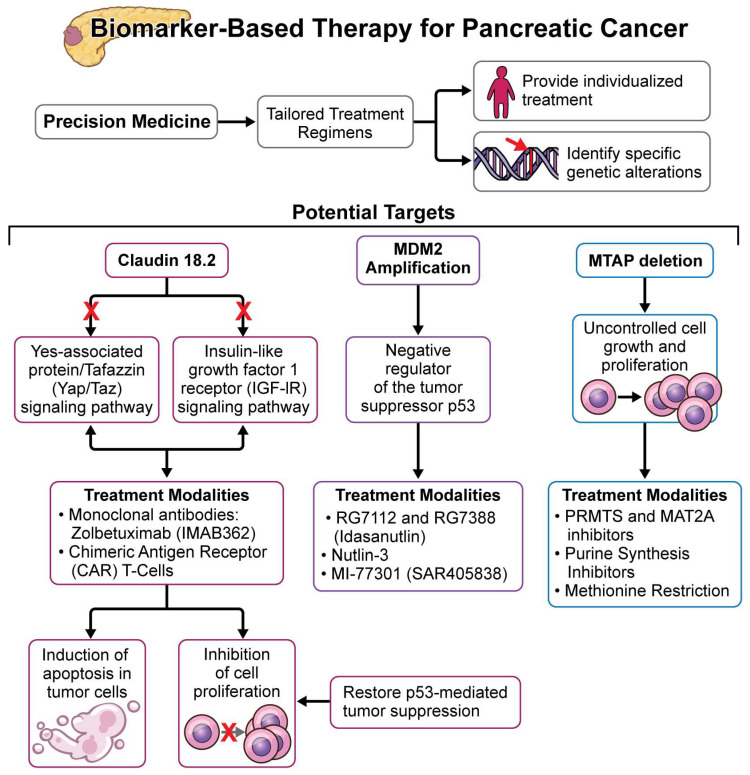
Schematic representation of the molecular classification of pancreatic cancer subtypes. This figure illustrates the categorization based on genetic mutations, gene expression profiles, and protein markers, which are critical for guiding treatment decisions and improving patient outcomes.

Among these challenges, precision medicine has emerged as promising hope, potentially offering a new approach to pancreatic cancer management [12]. By understanding the intricate molecular landscape of the disease, precision medicine holds promise for personalized interventions tailored to the unique genetic makeup of individual patients. We therefore aim and hope to gain a deeper understanding of the molecular classification of pancreatic cancer and apply next-generation sequencing (NGS) to identify unique genetic alterations for therapy, which could significantly benefit a subset of patients. Exploring DNA damage response (DDR) processes and their mutations in pancreatic cancer is a vibrant field of study. The development of pancreatic cancer is significantly associated with mutations in key DDR genes, such as BRCA-1 and BRCA-2 [13]. Additionally, recent progress has shown new possible approaches to treat pancreatic cancer, such as targeting MDM2 amplification, Claudin 18.2, and MTAP deletion [14,15,16]. These novel targets could be a great resource to enhance survival rates, especially in metastatic pancreatic cancer, ushering in a new era of therapeutic possibilities (Figure 1).

### 1.3. Biomarkers for Pancreatic Cancer

**Figure 2:**
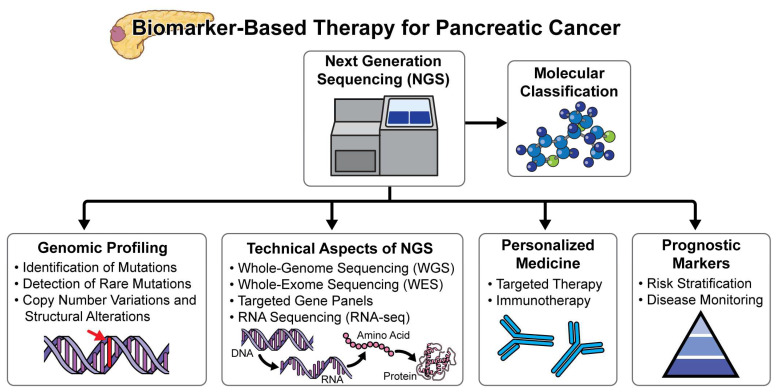
Schematic representation of the diagnostic and treatment strategies for pancreatic cancer. This diagram highlights the role of biomarkers in early detection, the use of molecular classification to guide personalized treatment, and the potential of next-generation sequencing (NGS) to identify unique genetic alterations for targeted therapies.

In various tissues and body fluids like blood, urine, saliva, and cyst fluid, biomarkers, which can be genes, RNA, proteins, or metabolites, serve as important indicators in pancreatic cancer [17]. Protein markers such as CA19-9 and carcinoembryonic antigen (CEA) found in the blood of pancreatic cancer patients can aid in diagnosis and treatment assessment. Biomarkers are also being looked at in terms of cell-free DNA, metabolome compounds, immune and stromal signatures, microbiome compositions, and microRNAs [18,19,20]. Liquid biopsy, encompassing exosomes, circulating tumor DNA (ctDNA), and circulating tumor cells (CTCs), is another area of interest [21,22]. Many aspects could be improved thanks to the emergence of genomic-based therapy. For example, personalized medicine for each patient, finding the disease early, monitoring treatment response, predicting recurrence, and selecting the right treatment based on biomarkers like BRCA1/2 mutations for Olaparib or microsatellite instability-high (MSI-high) for pembrolizumab [17,19,23]. Therefore, biomarkers present a promising resource for improving pancreatic cancer outcomes by contributing to early detection, personalized treatment, and effective monitoring of disease progression and treatment response (Figure 2).

### 1.4. Cancer Survival Rates

The survival rate of pancreatic cancer remains one of the lowest among major cancers, reflecting its aggressive nature and limited treatment options. Despite incremental improvements in survival rates over the years, the overall prognosis of patients with pancreatic cancer remains grim. The survival rates for pancreatic cancer differ significantly depending on the stage: when the cancer is localized, approximately 44% survive for five years. However, when the stage is more advanced as in the majority of diagnosed patients, the rate drops to 16%, and if it has metastasized to distant parts of the body, only 3% survive for five years, resulting in an overall five-year relative survival rate of 13% [24].

Significant challenges persist despite efforts to improve survival outcomes through advancements in treatment modalities. The complex molecular landscape and heterogeneity of pancreatic cancer present significant obstacles to the development of effective therapies. Additionally, the emergence of treatment resistance and disease recurrence exacerbated the challenges of managing pancreatic cancer.

This review not only discusses the highlights of the latest advancements in biomarker-based therapy for pancreatic cancer, inspired by Dr. Pishvaian’s Keynote Conference at MedNews Week, but also aims to provide an overview of the present literature on the topic [25]. While pancreatic cancer presents a plethora of challenges owing to its aggressive nature and limited treatment options, the emergence of biomarker-driven therapies offers a sparkle of hope. 

## 2. Present and Future of Precision Medicine in Pancreatic Cancer

In the context of pancreatic cancer management, precision medicine emerges as a promising assay, paving the way to personalized interventions tailored to the genetic makeup of individual patients [26,27]. At its core, precision medicine entails molecular testing with a therapeutic purpose, aiming to identify actionable biomarkers that present a significant predictive value in guiding treatment decisions. Precision medicine in the context of pancreatic cancer involves tailoring medical treatments based on an individual’s unique biology. This approach relies on biomarker testing of tumor tissue, which reveals the tumor’s specific genes and proteins. By understanding these molecular characteristics, doctors can therefore customize treatment approaches to target the tumor more effectively. Genetic testing is another crucial component of precision medicine [28]. It identifies inherited mutations that can impact how the tumor responds to treatment. Additionally, researchers have explored the use of patient-derived organoids (PDOs) for precision medicine in pancreatic cancer [26]. Researchers create PDOs from tissues obtained during surgical resection or biopsies, which enable ex vivo chemotherapeutic screening (pharmacotyping) and predict clinical therapeutic responses. Molecular profiling is also key, involving the analysis of genetic and molecular characteristics to identify effective treatment regimens [29]. This approach considers both genome-based medicine and individualized drug screening, aiming to improve outcomes for pancreatic cancer patients.

### 2.1. Molecular Classification and Precision Medicine

Molecular classification, or molecular subtyping, is not the same as precision medicine. Precision medicine extends beyond traditional molecular classification, which involves large-scale molecular characterization, often from large consortia of patient samples using gene sequencing and transcriptomics. Molecular classification involves categorizing diseases, such as cancer, based on specific molecular or genetic features like alterations in DNA, RNA, proteins, or other cellular components [30,31]. Its purpose is to identify distinct subtypes within a disease, allowing for more precise diagnosis and treatment tailored to specific patient groups. In pancreatic cancer, molecular classification helps identify different tumor subtypes based on genetic mutations, gene expression patterns, or protein markers, which can influence disease progression and treatment options.

Precision medicine, on the other hand, aims to provide individualized treatment based on a patient’s unique characteristics, including genetics, environment, lifestyle, and disease profile [32,33]. It goes beyond a one-size-fits-all approach by considering variability among patients and tailoring interventions accordingly. Precision medicine in pancreatic cancer involves biomarker testing to identify specific genetic alterations (e.g., mutations in BRCA1/2, PALB2) that guide treatment decisions, targeted therapies that specifically target molecular abnormalities (e.g., PARP inhibitors for patients with BRCA mutations), and leveraging immunotherapies based on individual immune profiles [34,35].

The key differences between molecular classification and precision medicine lie in their scope, clinical application, and level of personalization. Molecular classification focuses on understanding disease subtypes based on molecular features, informing diagnosis and prognosis. Precision medicine, on the other hand, encompasses a broader approach, considering individual patient characteristics for tailored treatment decisions aimed at improving outcomes. In summary, both concepts work together to optimize treatment strategies and improve outcomes in pancreatic cancer by understanding the disease’s diverse nature and translating this knowledge into individualized patient care.

### 2.2. Next-Generation Sequencing Efforts in Pancreatic Cancer

Next-generation sequencing (NGS) plays a crucial role in advancing our understanding of pancreatic cancer and guiding personalized treatment strategies [36]. NGS efforts identify these biomarkers by enabling a more comprehensive analysis of tumor genomes, revealing a diverse array of potential therapeutic targets. NGS efforts in pancreatic cancer have consistently revealed that at least 25% of pancreatic cancers harbor potentially actionable molecular biomarkers [37]. The majority of these biomarkers fall into the DNA damage response and repair (DDR) category or DDR mutations. Some patients, in small but repeatable groups, also show changes in microsatellite instability (MSI), different receptor tyrosine kinases, and different molecular signaling pathways [38,39,40,41]. These are potentially targetable, especially as our drugs to target those pathways are increasing in number. Pancreatic cancer is a highly lethal malignancy with technical challenges for genomic research. Traditional methods fall short due to the complexity of the disease. NGS allows comprehensive analysis of the entire genome, revealing somatic alterations unique to each tumor. By analyzing miRNA and specific proteins, oncologists can pinpoint therapeutic targets specific to pancreatic cancer [42]. NGS helps identify actionable alterations that guide treatment decisions. By tailoring therapies based on individual genomic profiles, NGS enables personalized approaches. This customization improves patient outcomes and offers hope for better survival rates. Combining NGS with machine learning allows deeper insights into tumor exomes [43]. Machine learning algorithms analyze vast genomic data, predict treatment responses, and guide precision medicine strategies. In summary, NGS empowers clinicians to unravel the genomic complexity of pancreatic cancer, identify potential therapeutic targets, and pave the way for personalized treatment options.

### 2.3. Remarkaable Benefit

Disproportionate benefit in pancreatic cancer refers to the significant advantages a specific intervention or treatment can provide for a particular subgroup of patients compared to others [44]. Cases, where appropriate therapy targets molecular abnormalities, exemplify this benefit, leading to treatment responses that significantly surpass the typical outcomes of standard cancer care. For instance, approximately one percent or slightly less of pancreatic cancers are MSI-high or have a mismatch repair deficiency [45]. When immunotherapy targets these tumors appropriately, patients often experience substantial benefits. 

Neoadjuvant treatment has emerged as a crucial strategy in the management of pancreatic cancer, particularly for patients with locally advanced disease or borderline resectable tumors. This approach involves administering chemotherapy or chemoradiotherapy before surgical resection. The rationale behind neoadjuvant therapy includes downsizing the tumor, potentially increasing the likelihood of achieving complete surgical resection (R0 resection), and addressing micrometastases early in the treatment course.

In pancreatic cancer management, disproportionate benefit plays a crucial role in several aspects:

1. Improved Survival with Surgery: Patients with pancreatic cancer who are eligible for surgery to remove the tumor can expect a significantly longer survival. Experts consider surgical resection as potentially curative, particularly when the tumor remains localized and has not spread beyond the pancreas [46]. According to recent studies, the median survival for patients undergoing successful surgical resection can be extended to approximately 20–23 months compared to a median of 6–11 months with non-surgical management. Moreover, advancements in surgical techniques and perioperative care have further improved outcomes, reducing postoperative complications and enhancing recovery rates [47].
2. Tailoring Therapies: While the majority of pancreatic cancer patients do not have germline mutations, using all available tools, including genetic testing, can help select therapies. Personalized treatment may not achieve a cure but can improve quality of life and extend survival [48,49]. For example, patients with BRCA mutations may benefit from PARP inhibitors, which have shown promise in prolonging progression-free survival. Additionally, tailored chemotherapeutic regimens based on genetic profiling can lead to better management outcomes. The implementation of molecular profiling in routine clinical practice allows for more precise targeting of therapies, potentially leading to better responses and fewer side effects compared to conventional treatments. The implementation of molecular profiling in routine clinical practice allows for more precise targeting of therapies, potentially leading to better responses and fewer side effects compared to conventional treatments. Precision medicine approaches, guided by biomarker or genetic testing, have demonstrated significant benefits in patient outcomes. Specifically, therapies matched to biomarkers or genetic profiles have been associated with extended survival rates (Pancreatic Cancer Action Network [PanCAN], 2024). PanCAN advocates for the adoption of genetic testing for inherited mutations at the time of diagnosis and recommends biomarker testing of tumor tissue for all patients, unless medically contraindicated. They provide comprehensive resources, including the Know Your Tumor^®^ precision medicine service, to facilitate informed treatment decisions and enhance patient care. Next-generation sequencing (NGS) is now widely utilized to detect diagnostic, prognostic, and predictive mutations across various cancers, contributing significantly to enhanced treatment efficacy [45,50,51]. In pancreatic cancer (PC), genomic profiling data have demonstrated potential benefits in guiding treatment decisions and improving patient survival [52,53]. Precision medicine, which tailors therapies based on molecular profiling of gene expressions and mutations, is anticipated to play a crucial role, especially in cases of unresectable pancreatic cancer. These mutations often involve genes such as KRAS, TP53, CDKN2A, and SMAD4, among others, which are known to play critical roles in pancreatic cancer pathogenesis and progression. The identification of these mutations through NGS holds promise for personalized treatment approaches, including targeted therapies and enrollment in clinical trials aimed at exploiting specific molecular vulnerabilities in pancreatic cancer cells. Despite challenges such as tumor heterogeneity and the complex genomic landscape of pancreatic cancer, NGS continues to emerge as a valuable tool in oncology.
3. Challenges: Regrettably, only approximately 10% of patients with pancreatic cancer receive an early diagnosis, thereby qualifying them for potentially curative surgical resection [50]. Most cases receive a diagnosis at a more advanced stage, which restricts the available treatment options. This highlights the need for improved early detection methods and public awareness to increase the rate of early diagnosis and intervention. Furthermore, the development of more effective systemic therapies for advanced-stage pancreatic cancer remains a critical area of ongoing research. Recent efforts in biomarker discovery and the utilization of liquid biopsies are promising steps toward earlier detection and better monitoring of treatment response.

### 2.4. Future Perspectives in Precision Medicine

Identifying and understanding the several benefits and potential of precision medicine in pancreatic cancer allows clinicians to tailor treatments, improve outcomes, and enhance the quality of life for patients with this challenging disease. By recognizing which subgroups of patients are likely to benefit the most from specific interventions, clinicians can make more informed decisions, optimizing the use of available therapies and predicting therapy response. This targeted approach therefore not only improves survival rates but also minimizes unnecessary side effects, making treatment more tolerable for patients [34]. Furthermore, the integration of advanced diagnostic tools and personalized medicine enables a more precise approach to cancer care. By taking advantage of genetic testing and molecular profiling, healthcare providers can identify actionable mutations and tailor therapies accordingly, leading to better management of the disease. This personalized approach is particularly crucial in pancreatic cancer, where the heterogeneity of the tumor often requires individualized treatment plans. The potential for disproportionate benefits also underscores the importance of continued research and innovation in pancreatic cancer treatment. As new therapies and diagnostic techniques are developed, the ability to identify patients who will benefit the most from these advancements will be essential. This will not only improve clinical outcomes but also contribute to a more efficient allocation of healthcare resources, ensuring that the most effective treatments are available to those who need them the most [34].

Ultimately, the goal is to move towards a more precise, patient-centered model of care that not only extends survival but also enhances the overall quality of life for patients with pancreatic cancer. This involves a multidisciplinary approach, combining the expertise of oncologists, surgeons, geneticists, and other healthcare professionals to provide comprehensive and effective care. As we continue to understand the unique benefits certain treatments can provide to specific patient populations, we can move one step closer to always more successful and personalized cancer therapies.

## 3. Advancing Pancreatic Cancer Treatment: The Power of Targeted Therapies and Genetic Insights

Identifying and understanding the plethora of benefits of targeted therapy in pancreatic cancer allows clinicians to tailor treatments, improve outcomes, and enhance the quality of life for patients with this challenging disease. By leveraging targeted therapies and advanced diagnostic tools, healthcare providers can indeed significantly impact patient prognosis, especially when specific genetic abnormalities are present. One critical area of focus in pancreatic cancer treatment is the role of genetic mutations in the DNA Damage Response (DDR) pathway.

DNA Damage Response (DDR) is an integral process in DNA replication that allows the body to identify damage done to the DNA and repair it. However, when the DDR process is altered or abnormal, the mutation can allow for damaged DNA to replicate without correction. From an oncological perspective, patients with a DDR abnormality are at a higher risk of developing cancer. Looking specifically at pancreatic cancer, it is found that approximately 17–25% of patients present mutations of a DDR gene [25]. These are specific to the homologous recombination pathway of the DDR gene (HR-DDR) [1]. HR-DDR mutations most notably occur in the BRCA-1, BRCA-2, ATM, PALB2, ATRX, and RAD51 genes [25].

BRCA-1 (Breast Cancer 1 Gene) and BRCA-2 (Breast Cancer 2 Gene) specifically account for 5–7% of mutations, yet up to 40% of patients with BRCA mutations do not have a family history of cancer [25]. This highlights the importance of germline testing to detect these mutations, irrespective of family history. These genes play crucial roles in DNA repair and homologous recombination, serving to suppress tumors by preventing the replication of damaged DNA [51]. Unfortunately, mutations in these genes can lead to a loss of tumor suppression function, allowing tumors to develop [51]. While BRCA mutations are traditionally associated with hereditary breast and ovarian cancers, recent studies have identified these mutations also in the context of pancreatic cancer [25,52].

In addition to BRCA mutations, other genes involved in the DDR pathway, such as ATM, PALB2, ATRX, and RAD51, are also implicated in pancreatic cancer susceptibility [25]. These genes play diverse roles in DNA repair processes, and mutations in any of them can contribute to pancreatic tumorigenesis. Understanding the specific mutations present in individual patients is crucial for guiding treatment decisions and improving outcomes. However, detecting these mutations solely based on family history could be a limitation and may lead to overlooking a significant portion of patients who could also benefit from targeted therapies. Furthermore, beyond genetic testing, advancements in treatment modalities have led to the development of targeted therapies tailored to specific genetic mutations. One such approach is platinum-based chemotherapy, which has shown efficacy in patients with DDR mutations [25,51]. This treatment works by eradicating tumor cells and preventing their further replication. However, platinum-based chemotherapy is associated with significant side effects, underscoring the need for alternative treatment options [52].

Poly (ADP-ribose) polymerase inhibitors (PARP inhibitors) represent a promising class of targeted cancer drugs that inhibit the enzyme poly ADP ribose polymerase (PARP), which is involved in DNA replication and repair [53]. Recent studies have demonstrated improved progression-free survival (PFS) in patients with BRCA mutations treated with PARP inhibitors [25,52]. However, the efficacy of PARP inhibitors may vary depending on the specific genetic alterations present in the tumor. Additionally, ongoing research is exploring combination therapies involving PARP inhibitors to enhance treatment outcomes and overcome potential resistance mechanisms. Despite their efficacy, PARP inhibitors are not without side effects. Common adverse reactions include fatigue, nausea, and anemia; however, these effects are generally manageable and do not significantly impact the patient’s health-related quality of life [52]. Several PARP inhibition drugs have either undergone clinical trials or received approval for tumor management. As of 2023, the Federal Drug Administration (FDA) has sanctioned four PARP-inhibitor drugs for clinical use: olaparib, rucaparib, niraparib, and talazoparib [54], primarily for monotherapy PARP-inhibition therapy treatment. Current clinical studies are exploring combination therapies involving PARP inhibitors to enhance patient outcomes and overcome potential resistance mechanisms [55]. These combination strategies include androgen deprivation, immunotherapy, cytotoxic chemotherapy, and radiation therapy [55]. The aim of these trials is to reduce the PARP inhibition resistance observed in some patients. While further research is required for conclusive evidence, preliminary results from these combination therapies are already promising, indicating potential avenues to overcome PARP-inhibition resistance. Currently, platinum chemotherapy stands as the primary treatment modality for patients with germline mutations in pancreatic cancer. However, this approach comes with notable drawbacks, including its association with severe and sometimes debilitating side effects. Recognizing the limitations of platinum chemotherapy, the integration of PARP inhibition therapy into treatment protocols emerges as an equally crucial consideration.

While the concurrent use of PARP inhibitors alongside platinum chemotherapy holds promise, recent studies have shed light on potential challenges. One study revealed the presence of a potential cross-resistance phenomenon when these treatments are administered sequentially. Despite this, a substantial proportion—over 40%—of patients still exhibited responsiveness to sequential treatment. These findings underscore the complexity of treatment interactions and emphasize the need for further research to understand optimal treatment sequencing strategies [56].

Moreover, the efficacy of PARP inhibition therapy is not uniformly positive across all patient subgroups. Variability in treatment response is often attributed to the diverse spectrum of DNA alterations present in pancreatic cancer. Consequently, identifying which patients would derive the most benefit from PARP inhibition therapy poses a significant clinical challenge. While PARP inhibition holds potential for certain patient subsets, the lack of information regarding its efficacy in specific pancreatic cancer subtypes underscores the imperative for further research. Clarifying the molecular underpinnings of treatment response will enhance the clinical reliability of PARP inhibition therapy and inform personalized treatment approaches tailored to individual patient profiles [57].

Therefore, while the integration of PARP inhibition therapy alongside platinum chemotherapy represents a promising weapon in pancreatic cancer treatment, challenges such as potential cross-resistance and variability in treatment response necessitate ongoing research efforts. Addressing these complexities will help to refine treatment strategies, optimize patient outcomes, and lead to more personalized and effective therapeutic approaches in the management of pancreatic cancer.

## 4. Emerging Therapeutic Targets in Metastatic Pancreatic Cancer: MDM2, CLAUDIN 18.2, and MTAP Deletion

In the field of oncology’s ever-expanding horizon, there is a plethora of novel emerging targets, particularly in the context of metastatic pancreatic cancer survival. MDM2 amplification plays a crucial role in disease progression by disrupting the regulatory balance between MDM2 and the tumor suppressor p53. This disruption promotes uncontrolled tumor growth and metastasis, contributing to the aggressive nature of the disease [15]. While MDM2 amplification is relatively rare in pancreatic cancer compared to other malignancies, its significance as a driver of disease aggressiveness highlights its potential as a therapeutic target [58]. Despite its low prevalence rates, MDM2 amplification presents a unique vulnerability for therapeutic aims, offering a promising strategy for combating this unfortunately nowadays deadly disease. 

In recent years, early clinical trials investigating MDM2 inhibitors have reported encouraging results in pancreatic cancer patients [59]. These inhibitors aim to restore p53-mediated tumor suppression and inhibit cancer cell proliferation, offering hope for patients with metastatic pancreatic cancer. While the outcomes of these trials are still evolving, initial findings showed tangible responses in select patient cohorts, highlighting the potential efficacy of MDM2-targeted therapies in improving clinical outcomes. Particularly, in de-differentiated liposarcomas, which share similarities with the aggressiveness of pancreatic cancer, MDM2 inhibitors have shown efficacy, hinting at their potential applicability in advancing also the treatment landscape for pancreatic malignancies [60]. One specific clinical trial, the Glow Trial, found that Zolbetuximab plus CAPOX significantly improved progression-free survival [PFS] and overall survival [OS] in patients that were diagnosed with claudin-18 isoform 2 [CLDN 18.2]-positive, HER2-negative, locally advanced unresectable or mG/GEJ adenocarcinoma [61]. It was found that adding Zolbetuximab to chemotherapy saw an approximately 31% reduction in disease progression and an approximately 25% reduction in disease-related death [61]. This study highlights the use of tumor-associated antigens (TAAs) and their indispensable use when it comes to treating gastrointestinal cancers [61]. 

As research progresses, further study of the mechanisms and prevalence of MDM2 amplification in pancreatic cancer, and ongoing clinical trials will provide valuable insights into optimizing the efficacy and safety of MDM2-targeted therapies [62]. Overall, MDM2 amplification represents a promising therapeutic target in the context of metastatic pancreatic cancer management [63]. By offering a novel approach to disrupt oncogenic signaling and restore tumor suppressor function, targeting MDM2 amplification appears to have the potential to improve outcomes for affected patients. Continued research and clinical exploration will be essential to fully realize the therapeutic potential of MDM2 inhibitors and to integrate them into the standard of care for metastatic pancreatic cancer.

Targeting CLAUDIN 18.2 has appeared as a promising therapeutic strategy, particularly in metastatic pancreatic cancer, owing to its pronounced expression in these tissue tumors. This tight junction protein therefore offers a tangible avenue for targeted interventions. The integration of CLAUDIN 18.2-targeted therapies, such as zolbetuximab, into standard chemotherapy regimens, has shown promising results in clinical trials. Notably, these trials have revealed improved progression-free and overall survival in CLAUDIN 18.2-overexpressed gastric cancers. High expression of CLAUDIN 18.2 in pancreatic cancer is crucial, as it is associated with initiation, progression, metastasis, and prognosis, making it a potential therapeutic target [64]. Clinical trials evaluating CLAUDIN 18.2-targeted therapies, including those for pancreatic and gastric cancers, are currently ongoing, though official results are pending. This research aims to understand efficacy data and potential side effects, adding a significant piece of the puzzle to the therapeutic landscape for metastatic pancreatic cancer. Furthermore, some additional studies have also been important to better characterize the function of this promising molecule. Zhou et al. demonstrated that Claudin 18.2 can inversely regulate the activity of Yes-associated protein (YAP), thereby promoting cell proliferation and tumorigenesis [65]. Additionally, Claudin 18.1 has been shown to inhibit Yes-associated protein/tafazzin (Yap/Taz) and insulin-like growth factor 1 receptor (IGF-1R) signaling pathways, ultimately resulting in AKT inhibition [66,67]. These findings therefore emphasize the pivotal role of Claudins as important signaling centers involved in cell proliferation and tumorigenesis [68].

Recently, other potential candidate targets have also emerged, further enhancing interest and hope in the field. MTAP deletion, prevalent in 20% to 30% of pancreatic cancer cases, is a significant anomaly intricately linked to the disease’s progression. Previous research has unveiled the grim prognosis associated with MTAP deficiency in pancreatic ductal adenocarcinoma (PDAC). Expanding on this understanding, bioinformatics analysis of The Cancer Genome Atlas (TCGA) data has described a distinct signature of heightened glycolysis in PDACs lacking MTAP. The currently enigmatic role of MTAP deletion appears to have promising and profound therapeutic implications for metastatic pancreatic cancer [69]. Within this complex pathway, loss of the MTAP enzyme compromises cellular vulnerability, rendering cancer cells susceptible to targeted interventions. Through inhibition of MAT2A or PRMT5, exploiting the synthetic lethality cascade, novel therapeutic modalities have emerged, holding promise for cancers presenting MTAP deletion, including metastatic pancreatic malignancies. Clinical trials investigating PRMT5 and MAT2A inhibitors have demonstrated encouraging outcomes, highlighting the potential of synthetic lethality-based strategies in reshaping the treatment algorithm [70]. These findings therefore offer significant hope to patients, suggesting a promising shift towards more effective and targeted therapeutic modalities.

The convergence of innovative targets and precision medicine opens a new era in the management of metastatic pancreatic cancer. By moving beyond the one-size-fits-all approach, personalized therapies tailored to individual molecular profiles offer the potential to significantly extend survival and improve patient outcomes [71]. Dr. Pishvaian’s Keynote emphasizes the need to embrace both innovation and precision to be able to aim for improved metastatic pancreatic cancer survival. As emerging targets like MDM2 amplification, CLAUDIN 18.2-targeted therapies, and MTAP deletion become integrated into clinical practice, personalized medicine is set to revolutionize the management of this currently lethal disease. This not only brings hope to patients but also challenges the oncology community to pursue further research in the field. Through genomic profiling and targeted interventions, the promise of prolonged survival and improved quality of life is becoming a tangible reality for patients. 

The oncology community eagerly stands at the threshold of a new era, where precision and innovation drive the fight against cancer, with the potential of success also in the fight against the most advanced stages of this lethal disease.

## 5. Ongoing Advancements in Pancreatic Cancer Management through Biomarker-Based Clinical Trials and Precision Medicine

Biomarker-based clinical trials and therapeutic strategies therefore represent a groundbreaking advancement in the management of pancreatic cancer, aiming to optimize treatment outcomes through personalized approaches. These innovative trials employ biomarkers to identify patient subpopulations most likely to benefit from specific therapeutic interventions, including targeted therapies, immunotherapies, and combination regimens. By integrating molecular profiling into clinical trial design, basket trials, umbrella trials, and adaptive trial designs enables the customization of treatment regimens tailored to individual patients’ molecular profiles [72,73].

Currently, several clinical trials are underway to explore the efficacy of biomarker-driven therapies in the context of pancreatic cancer. 

For instance, the NCI-MATCH (Molecular Analysis for Therapy Choice) trial is a nationwide precision medicine cancer treatment clinical trial that assigns patients to targeted therapy based on the specific mutations identified in their tumors rather than their cancer type [74]. Another notable example is the TAPUR (Targeted Agent and Profiling Utilization Registry) study, which evaluates the safety and efficacy of FDA-approved targeted therapies in patients with advanced cancer harboring specific genetic alterations [75]. In addition, the COMPASS (Comprehensive Molecular Characterization of Advanced Pancreatic Ductal Adenocarcinoma for Better Treatment Selection) trial focuses on utilizing comprehensive molecular profiling to guide treatment decisions for patients with advanced pancreatic cancer. This trial aims to identify actionable mutations and stratify patients into subgroups that may benefit from specific targeted therapies or clinical trial enrolment [76]. The IMPaCT (Integrative Molecular Profiling of Pancreatic Cancer Therapy) trial is another example, aiming to match patients with targeted therapies based on the molecular characteristics of their tumors, allowing for more personalized treatment regimens [77].

Combining immunotherapy with targeted therapy seems to be a promising approach, especially in the treatment of metastatic pancreatic cancer, as evidenced by several relevant studies. The KEYNOTE-158 Phase II study involved 22 patients with microsatellite instability-high (MSI-H) or mismatch repair-deficient (dMMR) tumors treated with pembrolizumab, resulting in an 18.2% response rate (RR), 2.1 months of progression-free survival (PFS), and 4 months overall survival (OS) [78]. The CODEBREAK-100 Phase Ib/II study included 38 patients with KRAS G12C mutations treated with sotorasib, showing a 21% RR, 4 months PFS, and 6.9 months OS [79]. In the KRYSTAL-1 Phase II study, 21 patients with KRAS G12C mutations received adagrasib, achieving a 33.3% RR, 5.4 months PFS, and 8 months OS [80]. Schram’s 2021 Phase II study focused on 10 patients with NRG1 fusions treated with zenocutuzumab, yielding a 40% RR [81]. Sacher’s 2023 Phase I study reported a 36% RR for 22 patients with KRAS G12C mutations treated with diravasib [82]. Li’s 2024 Phase I/II study on 24 patients with KRAS G12C mutations treated with olomorasib demonstrated a 33% RR [83]. Finally, Hollebecque’s 2024 Phase I/II study involving 28 patients with KRAS G12C mutations treated with glecirasib reported a 46.4% RR and 5.5 months PFS [84].

Also, the National Cancer Institute (NCI) is supporting a broad range of research programs aimed at addressing pancreatic cancer more effectively, through different approaches. These programs range from basic research exploring the biological underpinnings of cancer to clinical research seeking to translate these findings into improved patient outcomes [76]. For example, the Pancreatic Cancer Cohort Consortium involves more than a dozen prospective epidemiologic cohort studies investigating the causes and natural history of pancreatic cancer. This consortium includes the genome-wide association study (GWAS) known as PanScan. The Pancreatic Cancer Detection Consortium (PCDC) focuses on developing and testing biomarkers for early-stage pancreatic cancer detection and identifying high-risk individuals.

The Pancreatic Ductal Adenocarcinoma (PDAC) Stromal Reprogramming Consortium (PSRC) is a multidisciplinary community of researchers bridging biological research with preclinical and translational research. Their goal is to identify and evaluate elements in the tumor microenvironment that drive PDAC progression and response to therapy. Additionally, the Pancreatic Specialized Programs of Research Excellence (Pancreatic SPOREs) are designed to quickly move basic scientific findings into clinical settings, supporting new approaches to the prevention, early detection, diagnosis, and treatment of pancreatic cancer. The RAS Initiative aims to understand mutations in RAS genes, which are implicated in over 90% of pancreatic cancers, to develop effective new therapies for RAS-related cancers.

Predictive biomarkers such as genetic mutations (e.g., KRAS, BRCA1/2), gene expression profiles, and protein markers (e.g., CA 19-9) guide patient stratification, facilitating the selection of optimal therapeutic agents [72,85,86]. 

Targeting KRAS mutations indeed represents a significant advancement in the treatment of PDAC. KRAS mutations, which occur in over 90% of PDAC cases, drive oncogenic signaling pathways that promote tumor growth and survival. Recent therapeutic strategies that specifically inhibit mutant KRAS have shown promising preclinical results, potentially transforming the therapeutic landscape for this aggressive cancer. The development of direct KRAS inhibitors, such as sotorasib (AMG 510) and adagrasib (MRTX849), has demonstrated efficacy in early clinical trials, providing new hope for patients with KRAS-mutant PDAC [87]. These breakthroughs highlight the potential of precision medicine to significantly improve outcomes.

However, the successful integration of biomarker-driven approaches into clinical trials faces several obstacles such as assay standardization, patient heterogeneity, and regulatory considerations. Overcoming these challenges requires collaborative efforts between researchers, clinicians, and regulatory agencies to establish robust biomarker validation processes and streamline their incorporation into clinical practice.

Despite these challenges, biomarker-based clinical trials hold promise for improving treatment efficacy, enhancing patient outcomes, and advancing the field of pancreatic cancer therapeutics. Recent research has highlighted the pivotal role of the ERK pathway in KRAS-mutated cell lines, revealing that its activation is crucial for tumor growth and survival, whereas the MAP pathway appears to be less significant [88]. These studies suggest that targeting the ERK pathway could yield more effective therapeutic strategies. The continued development and refinement of these approaches are essential for realizing the full potential of precision medicine in this challenging disease (Table 1).

## 6. Conclusions and Future Perspectives

As a significant step towards personalized medicine, incorporating biomarker testing into the treatment strategy for pancreatic cancer aligns treatment choices with the unique genetic profiles of individual patients. 

Several studies demonstrating the effects on patient survival highlight the significance of biomarker testing in pancreatic cancer management. As one of the many examples, in the clinical trial involving 1856 patients referred to the Know Your Tumor (KYT) program, 58% received personalized reports based on molecular testing. Among these, those with actionable alterations who received matched therapy showed significantly longer median overall survival compared to those receiving unmatched therapies (2.58 years vs. 1.51 years; HR 0.42, *p* = 0.0004). These results highlight the importance of personalized treatment guided by molecular testing in improving overall survival among pancreatic cancer patients [89].

The KRAS gene mutation is identified in 90% of PDAC patients, facilitating tumorigenesis through the MAP kinase pathway. Strategies to target the most common KRAS mutation, KRAS G12D, with specific inhibitors have shown limited efficacy. Recent studies indicate a correlation between RAS mutations and immunotherapy resistance, leading to observations that combining inhibitors with immunotherapy yields favorable outcomes. Inhibition of KRAS stimulates the Fas pathway, inducing cancer cell death and enhancing the presence of T cells while reducing myeloid cells in tumors [90]. However, tumors eventually recur, highlighting the need for a combination of more effective therapies with immune checkpoint inhibitors for sustained tumor regression and improved survival [91]. Pancreatic cancer patients without KRAS mutations exhibit other actionable genetic alterations, such as MSI/dMMR, BRAF mutations, and kinase-fusion genes, emphasizing the role of precision oncology in identifying potential targets for tailored treatments [92].

Tumor mutational burden (TMB) is also emerging as a biomarker to predict response to immunotherapy with immune checkpoint inhibitors (ICIs). In pancreatic cancer, just over 1% of patients exhibit a TMB-H phenotype, with about 60% of these having microsatellite instability (MSI-H). Hence, its role in pancreatic cancer remains less clear due to its low prevalence. Continued research and consensus on TMB cut-off values are therefore necessary to optimize its application in clinical settings [93].

A few other new strategies are also currently being explored. A phase 1 clinical trial recently assessed a chemoradiation regimen with nab-paclitaxel, capecitabine, and radiation for pancreatic cancer. The maximum tolerated dose of nab-paclitaxel was established at 75 mg/m^2^. Among 23 patients, no grade 3 or 4 toxicities were seen at the lower doses, but 5 patients at the maximum tolerated dose experienced grade 3 toxicities. Seven patients underwent surgical resection. Median overall survival was 21.2 months, and progression-free survival was 8.1 months. A more defined tumor interface response (IR) was associated with better survival outcomes. These promising results therefore support the development of further trials and suggest IR could be a meaningful biomarker [94].

Another study evaluated serum albumin (b-alb) as a biomarker for bevacizumab in advanced pancreatic cancer. Data from 264 patients showed that those with normal b-alb (≥3.4 g/dL) treated with bevacizumab had significantly better outcomes: median overall survival of 10.2 months versus 4.1 months (*p* = 0.0001) and median time to disease progression of 6.2 months versus 3.7 months (*p* = 0.0488). These results suggest that normal b-alb can predict benefits from bevacizumab, which is essential to select patients based on b-alb levels for better treatment outcomes [95].

Genetic variations combined with traditional tumor markers can improve pancreatic cancer detection. Incorporating single-nucleotide polymorphisms (SNPs) associated with CA19-9, CEA, and CA-125 levels increased sensitivity in identifying pancreatic ductal adenocarcinoma (PDAC) patients. However, combining markers slightly reduced specificity, suggesting that SNP information could enhance diagnostic accuracy for pancreatic cancer, potentially aiding in early detection and treatment decisions [96].

Germline BRCA testing and treatment strategies significantly impact health outcomes in metastatic pancreatic cancer patients. Among patients with gBRCAm, almost twice as many received platinum-based regimens with early testing strategies compared to no early testing (78.7% vs. 40.2%). Health outcomes, measured by progression-free life years (PF LYs), overall life years (LYs), and quality-adjusted life years (QALYs), were significantly higher in the early testing with available olaparib treatment scenario compared to other strategies (PF LYs: 1.27 vs. 0.55–0.87; LYs: 1.82 vs. 0.95–1.27; QALYs: 1.15 vs. 0.73–0.92) [97].

Despite the potential advantages of biomarker testing, its widespread utilization in clinical practice presents several challenges. One major obstacle is the low rate of treatment initiation among pancreatic cancer patients, with only 45% of patients receiving any form of treatment after diagnosis. This therefore leads to the need to improve awareness, access, and acceptance of biomarker-guided therapies among patients and healthcare providers alike. Additionally, there is a discrepancy in the uptake of germline testing, with only 30–50% of patients undergoing testing for inherited genetic mutations. Managing these barriers requires multiple efforts to enhance testing accessibility, streamline testing protocols, and integrate genetic counseling into routine care. To overcome these challenges and enhance the routine implementation of biomarker testing in pancreatic cancer care, several measures have been recommended. Reflexive Next-Generation Sequencing (NGS) and RNA testing should be evaluated for all pancreatic cancer patients at the time of diagnosis. Similar to standard practices in breast cancer care, where testing for hormone receptor status and HER2 expression is reflexive, labeling pancreatic cancer specimens as “likely pancreatic cancer” could prompt automatic biomarker testing, ensuring timely and comprehensive analysis of genetic alterations. Additionally, universal germline testing should be incorporated as a standard practice for all pancreatic cancer patients, consistent with existing guidelines recommending genetic evaluation for hereditary cancer syndromes. Systems should be established to facilitate the collection of blood or saliva samples for germline testing at the initial patient visit, with cascade testing strategies utilized to identify at-risk family members and enable early intervention.

Role of Immunotherapy in Pancreatic Cancer. Pancreatic adenocarcinoma is still one of the deadliest types of cancer worldwide, highlighting the need for better treatment choices. While immunotherapy has revolutionized treatment paradigms in various malignancies with significant survival benefits, pancreatic cancer has largely remained refractory to these advances. Current treatments primarily rely on chemotherapies, which offer modest benefits. Because pancreatic malignancies are immune-cold tumors, clinical trials of immunotherapies have shown a limited response [98].

The immune-cold nature of pancreatic ductal adenocarcinoma (PDAC) is attributed to its dense desmoplastic stroma, a physical barrier to immune cell infiltration, and its immunosuppressive microenvironment. Regulatory T cells, tumor-associated macrophages, and cancer-associated fibroblasts collaborate to create this immunosuppressive milieu, which inhibits effective immune responses [99]. Consequently, immune checkpoint inhibitors (ICIs) have demonstrated minimal improvements in patient outcomes. However, a subset of patients does benefit from these therapies, underscoring the need for predictive biomarkers and a deeper understanding of resistance mechanisms [98].

For patients with mismatch repair deficiency or microsatellite instability (MSI) who have pancreatic cancer, only anti-programmed cell death 1 (PD-1) antibodies have FDA approval. Despite this approval, the broader application of immunotherapy in pancreatic cancer remains challenging due to its overall low tumor mutational burden and complex tumor microenvironment [99,100].

A variety of current clinical trials are investigating different immunotherapy strategies, including combinations with other immunotherapy agents, targeted treatments, stroma-altering drugs, and chemotherapy. In PDAC patients with MSI-high status, for instance, pembrolizumab has shown notable radiographic responses [101]. Anti-PD-1 is being investigated intensively by researchers in conjunction with CAR T cells, pathway inhibitors, other immune checkpoint inhibitors, and cancer vaccines [102,103]. These combination treatments seek to increase T-cell recruitment and improve immune checkpoint inhibitor efficacy [99].

Cutting-edge immunotherapy techniques are currently under investigation, such as the utilization of oncolytic viruses, modification of T-cell receptors, the application of CAR T-cell therapy, the use of CAR natural killer cells, and the stimulation of cytokine-induced killer cells. These strategies are specifically developed to address various components of the tumor microenvironment (TME) and to tackle the obstacles posed by immunosuppression [104]. In addition, advanced methods such as single-cell sequencing and multi-omics analysis are being used to gain a deeper understanding of the composition of immune cells in pancreatic ductal adenocarcinoma (PDAC). This research has the potential to pave the way for the creation of innovative and powerful immunotherapies [104].

Despite the promising preclinical results, significant challenges remain. The dense fibrotic TME and immune escape mechanisms continue to limit the effectiveness of immunotherapies in clinical settings. Refining existing immunotherapies and validating novel targets through human clinical trials is crucial. The incorporation of advanced research fields like machine learning, artificial intelligence, and CRISPR/Cas-based technologies presents new opportunities for revolutionary advancements in immunotherapy for pancreatic cancer [104,105,106].

In conclusion, while the current role of immunotherapy in pancreatic cancer is limited, ongoing research and innovative approaches hold promise for future advancements. Continued efforts to elucidate the mechanisms of immune resistance and develop effective combination therapies are essential to harness the potential of immunotherapy in improving outcomes for pancreatic cancer patients.

Cancer-associated fibroblasts (CAFs) as a potential treatment target. Over the past few years, there has been a lot of focus on the role of cancer-associated fibroblasts (CAFs) in tumorigenesis. CAFs, an essential element of the tumor microenvironment, have a significant impact on advancing cancer growth and resistance to treatment through a range of mechanisms. Research has indicated that CAFs and cancer cells have a complex relationship, where they influence each other in a way that promotes tumor growth, angiogenesis, metastasis, and resistance to therapy [107,108,109,110].

CAFs have been implicated in the initiation of epithelial tumor formation and are known to release regulatory factors that support tumor growth and metastasis [107]. They exhibit pro-tumorigenic functions, modulating the tumor microenvironment through diverse mechanisms and supporting tumor progression [108]. Despite their heterogeneity and plasticity, CAFs are considered a potential target for cancer treatment, with preclinical and clinical trials suggesting their importance in solid tumors [107,108,109,111].

Targeting CAFs as a therapeutic strategy has shown promise in preclinical studies. Recent advances in therapies targeting cancer-associated fibroblasts (CAFs) include DNA-based vaccines, anti-CAF CAR-T cells, and strategies for modifying and reprogramming CAF functions. These approaches are being explored in clinical trials to determine their efficacy in treating various cancers by targeting the supportive role CAFs play in tumor growth and progression. However, translating these findings into clinical practice remains a challenge [108]. Various approaches, including reducing, eliminating, or reprogramming CAFs, have been explored, but their effectiveness in human clinical trials against different cancer types, including melanoma, breast cancer, pancreas cancer, and colorectal cancers, has been limited [108]. Challenges such as CAF heterogeneity, lack of specific target markers, and off-target effects need to be addressed to develop safe and effective CAF-targeting therapies [108,109,111].

Despite these challenges, understanding the biology of CAFs and their interactions with cancer cells provides valuable insights for developing novel therapeutic strategies. Targeting CAFs could potentially improve cancer treatments by disrupting the tumor-stroma crosstalk and enhancing the efficacy of existing therapies [109,110]. In conclusion, CAFs represent a promising target for cancer therapy, with their role in tumorigenesis and therapy resistance increasingly recognized. Further research is needed to unravel the complexities of CAF biology and develop effective CAF-targeting therapies for improving cancer treatment outcomes.

In conclusion, biomarker testing represents a crucial aspect of personalized medicine in pancreatic cancer care, offering the potential to tailor treatment strategies based on individual genetic profiles. With further research and the integration of biomarker testing into routine clinical practice and addressing existing barriers through targeted interventions, healthcare providers can optimize treatment decisions, improve patient outcomes, and ultimately enhance the quality of care for pancreatic cancer patients at all stages.

## Figures and Tables

**Table 1:** Summary of the ongoing biomarker-based clinical trials and precision medicine in pancreatic cancer management.

Clinical Trial Name	Aim and Description
NCI-MATCH (Molecular Analysis for Therapy Choice) [74]	Targets therapy based on tumor mutations rather than their cancer type.
TAPUR (Targeted Agent and Profiling Utilization Registry) [75]	Evaluates the safety and efficacy of FDA-approved targeted therapies in patients with advanced cancer harboring specific genetic alterations.
COMPASS (Comprehensive Molecular Characterization of Advanced Pancreatic Ductal Adenocarcinoma for Better Treatment Selection) [76]	Uses molecular profiling for advanced pancreatic cancer treatment.
IMPaCT (Integrative Molecular Profiling of Pancreatic Cancer Therapy) [77]	Matches patients with therapies based on tumor molecular characteristics.
KEYNOTE-158 (Phase II) [78]	Pembrolizumab in MSI-H/dMMR tumors: 18.2% RR, 2.1m PFS, 4 m OS.
CODEBREAK-100 (Phase Ib/II) [79]	Sotorasib in KRAS G12C mutations: 21% RR, 4 m PFS, 6.9 m OS.
KRYSTAL-1 (Phase II) [80]	Adagrasib in KRAS G12C mutations: 33.3% RR, 5.4 m PFS, 8 m OS.
Schram’s 2021 Phase II [81]	Zenocutuzumab in NRG1 fusions: 40% RR.
Sacher’s 2023 Phase I [82]	Diravasib in KRAS G12C mutations: 36% RR.
Li’s 2024 Phase I/II [83]	Olomorasib in KRAS G12C mutations: 33% RR.
Hollebecque’s 2024 Phase I/II [84]	Glecirasib in KRAS G12C mutations: 46.4% RR, 5.5 m PFS.I
Pancreatic Cancer Cohort Consortium [76]	Studies cause and natural history of pancreatic cancer (includes PanScan).
Pancreatic Cancer Detection Consortium (PCDC) [76]	Develops/tests early detection biomarkers.
Pancreatic Ductal Adenocarcinoma (PDAC) Stromal Reprogramming Consortium (PSRC)	Studies tumor microenvironment in PDAC progression and therapy response.
Pancreatic Specialized Programs of Research Excellence (Pancreatic SPOREs)	Translates basic research into clinical settings.
RAS Initiative [87]	Develops therapies for RAS mutations in pancreatic cancer.
